# Online distribution channel increases article usage on Mendeley: a randomized controlled trial

**DOI:** 10.1007/s11192-017-2438-3

**Published:** 2017-06-22

**Authors:** Paul Kudlow, Matthew Cockerill, Danielle Toccalino, Devin Bissky Dziadyk, Alan Rutledge, Aviv Shachak, Roger S. McIntyre, Arun Ravindran, Gunther Eysenbach

**Affiliations:** 1 Department of Psychiatry, Clinician-Investigator Program, University of Toronto, Toronto, ON Canada; 2 Institute of Medical Science, University of Toronto, Toronto, ON Canada; 3 Data Science Team, TrendMD Inc., MaRS Discovery District, West Tower, 661 University Avenue, #465, Toronto, M5G 1M1 ON Canada; 4 Institute of Health Policy, Management and Evaluation, University of Toronto, Toronto, ON Canada; 5 University of Toronto, Toronto, ON Canada; 6 Mood Disorders Psychopharmacology Unit, University Health Network, Toronto, ON Canada; 7 Centre for Addiction and Mental Health, Toronto, ON Canada; 8 Department of Psychiatry, University of Toronto, Toronto, ON Canada; 9 Centre for Global eHealth Innovation, Techna Institute, University Health Network, Toronto, ON Canada

**Keywords:** Bibliometrics, Mendeley, Randomized controlled trial, Article usage, Academic journals, Impact, TrendMD, Knowledge dissemination

## Abstract

Prior research shows that article reader counts (i.e. saves) on the online reference manager, Mendeley, correlate to future citations. There are currently no evidenced-based distribution strategies that have been shown to increase article saves on Mendeley. We conducted a 4-week randomized controlled trial to examine how promotion of article links in a novel online cross-publisher distribution channel (TrendMD) affect article saves on Mendeley. Four hundred articles published in the *Journal of Medical Internet Research* were randomized to either the TrendMD arm (*n* = 200) or the control arm (*n* = 200) of the study. Our primary outcome compares the 4-week mean Mendeley saves of articles randomized to TrendMD versus control. Articles randomized to TrendMD showed a 77% increase in article saves on Mendeley relative to control. The difference in mean Mendeley saves for TrendMD articles versus control was 2.7, 95% CI (2.63, 2.77), and statistically significant (*p* < 0.01). There was a positive correlation between pageviews driven by TrendMD and article saves on Mendeley (Spearman’s rho *r* = 0.60). This is the first randomized controlled trial to show how an online cross-publisher distribution channel (TrendMD) enhances article saves on Mendeley. While replication and further study are needed, these data suggest that cross-publisher article recommendations via TrendMD may enhance citations of scholarly articles.

## Background

As global research output continues to increase, the competition for readers’ attention amongst scholarly publishers, journals, and, authors is becoming tougher. Traditionally, scholarly publishers promoted issues of journals containing multiple articles, but with the increasing dominance of electronic publishing, there is growing interest in promoting individual articles (Fox et al. 2016a). It remains common practice for authors to promote their research articles by presenting at conferences; however there is scant evidence to suggest that such tactics are actually effective at enhancing scholarly article impact (de Leon and McQuillin 2014). Many journals engage in online tactics, such as promoting scholarly article links in social media channels to attract and engage readers. There is robust data on the effectiveness of online advertising [i.e. Google AdWords (Turnbull and Bright 2008) and social media (Hollis 2005)] for driving purchases of consumer goods and building brands (Tiago and Veríssimo 2014). However, there is a paucity of data on the efficacy of social media and other online channels to distribute scholarly content and drive impact of individual articles (Fox et al. 2016b).

Prior research has yielded inconclusive results as to whether social media can enhance pageviews and/or article impact (Fox et al. 2014, 2016b; Dixon et al. 2015; Thoma et al. 2015; Hand et al. 2016). For example, a study completed in 2014 by Fox et al. (2014), found no differences in median 30-day pageviews for articles randomized (*n* = 121) to a social media promotion strategy that involved articles receiving posts on Twitter and Facebook containing a toll-free link to the full-text version of the article. A key limitation, however, was that investigators did not examine the effects of paid tweets or sponsored Facebook posts on article pageviews.

The Fox et al. study stirred controversy by some groups in the medical publishing community, citing that the social media strategy was not intensive enough, and that their conclusions were not generalizable (Dixon et al. 2015; Thoma et al. 2015). In their response letter, Thoma et al. (2015) cited their experience running a comprehensive social media campaign, which lead to a 289% increase in traffic to the *Annals of Emergency Medicine* when compared with the prior calendar year. However, as Fox et al. (2016b) pointed out, these data are from an ecological association, and the traffic increase cannot be attributed to the social media campaign based on the observational study design. The increase in traffic could have been due to organic changes in traffic to the journal, rather than the social media campaign. In another response letter, Dixon et al. (2015) found that article pageviews increased from 3234 to 6768 in the 7 days following the posting of a blog article on Radiopaedia.org containing a summary of a manuscript. These data, however, are potentially confounded by selection bias; did the blog post lead to enhanced visibility, or was the manuscript blogged about noteworthy to begin with?

To address the concerns, Fox et al. (2016b) completed a follow-on randomized controlled trial (*n* = 152 articles; 74 social media; 77 control) in 2016 that utilized a more intensive social media promotion strategy. Investigators retweeted posts of articles on Twitter to encourage online interaction. To increase the viewership on Facebook, investigators sponsored Facebook posts for 24 h for a total of $10 USD for each post. Despite the increased intensity of social media, there were no differences in 30-day article pageviews between intervention (499.5 median pageviews) and control (450.5 median pageviews) (Fox et al. 2016b). These data however, are still limited by the fact that paid Tweets were not used, and that no other article metrics were reported aside from 30-day pageviews, which have not been found to strongly correlate to future impact (Perneger 2004).

The choice of what metrics to use when assessing the effectiveness of online distribution tactics is an area of active research. Though citation counts remain the gold standard of measuring scholarly article impact, citations take a long time to accrue on articles, and are therefore not well suited to assess the immediate impact of distribution tactics. Several studies have examined early indicators of impact, known as “altmetrics” (Eysenbach 2011; Li et al. 2011; Thelwall et al. 2013; Fox et al. 2016a). Altmetrics include the number of times a journal article is viewed (pageviews), downloaded, mentioned, or discussed on social media, or saved by various citation manager programs such as Mendeley. The Almetric score is a popular article impact metric that reflects aggregate mentions of articles on many social media channels (e.g. Twitter, Facebook, etc.), Wikipedia, news, and blogs (Altmetric 2016). However, the literature on the relationship between Altmetric scores and traditional measures of impact, such as citations is mixed. One study suggests that tweets of articles on Twitter correlate to citations (Eysenbach 2011). In contrast, other studies have not found a relationship between tweets or Altmetric scores to citations (Priem et al. 2012; Thelwall et al. 2013).

Replicated studies have found that the most robust early predictor of citations is article saves/reader counts of scholarly articles on reference managers, such as Mendeley (Priem et al. 2012; Lin and Fenner 2013; Zahedi et al. 2014, 2015; Ebrahimy et al. 2016; Maflahi and Thelwall 2016; Thelwall and Wilson 2016; Li and Thelwall 2012). This makes intuitive sense; as a common practice, scholars save articles in bibliographic software such as Mendeley in advance of creating other work (i.e. during literature reviews) (Pautasso 2013). Accordingly, a 2014 study revealed that 63% of Web of Science articles from 2005 to 2011 had at least one Mendeley save by April 2013 (Zahedi et al. 2014) and found a moderate Spearman Rho correlation (*r* = 0.49) between Mendeley saves and citation counts (Zahedi et al. 2014). These findings were replicated by a recent large systematic review of 90,728 articles published in 7 PloS journals between 2009 and 2013. The study, which utilized a path analysis method to assess causal relationships, found that Mendeley article saves preceded, and explained 69% of the variance in article citation counts (Ebrahimy et al. 2016). Investigators found that visibility (as measured by pageviews and article downloads) was necessary, but not sufficient to drive article saves on Mendeley (Ebrahimy et al. 2016). In other words, the more an article is seen, the higher the probability for it to be saved in Mendeley; but many articles with high pageview and download counts did not go on to be highly saved on Mendeley or cited. In contrast, the study (Ebrahimy et al. 2016) found that other altmetrics such as article mentions on Twitter, F1000 recommendations, Facebook posts, and Altmetric scores were not predictive of future citations (Ebrahimy et al. 2016). These data were largely consistent with other observational data suggesting article saves on Mendeley is the best early predictor of future citations (Priem et al. 2012; Thelwall and Wilson 2016).

Notwithstanding, the primary issue facing scholarly content producers is that there are currently no evidenced-based strategies that have been shown to enhance article saves on Mendeley. We previously reported that distribution of article links in the cross-publisher content recommendation network (TrendMD), augment pageviews (Kudlow et al. 2016); however, we do not know if this increased visibility affects article Mendeley saves. The purpose of this study was to examine the impact of distributing article links in a cross-publisher recommendations network on article saves on Mendeley.

## Methods

**Fig. 1:**
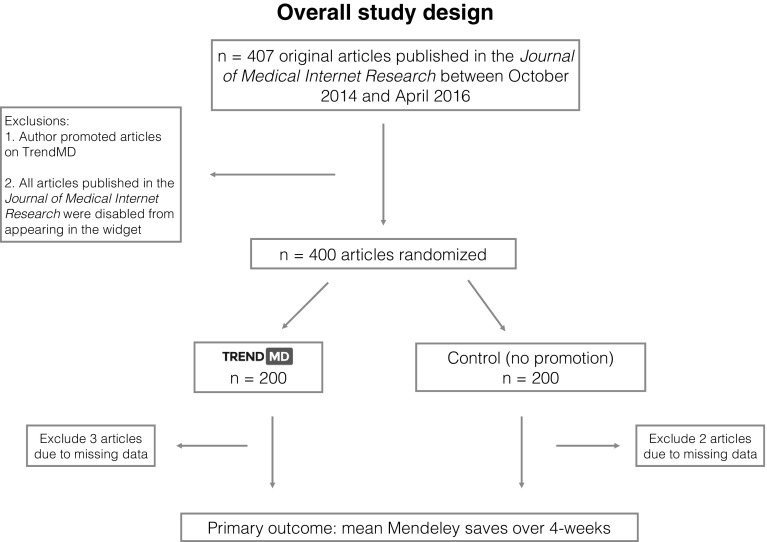
Overall study design

We conducted a 4-week randomized controlled trial that included 400 Open Access articles published in the *Journal of Medical Internet Research (JMIR)* between October 1 2014 and April 30 2016. JMIR is a leading health informatics and health services/health policy journal (ranked first by impact factor in these disciplines). It focuses on emerging technologies in health, medicine, and biomedical research (Harriman 2004). We selected articles published between 6 months and 2 years prior to the beginning of the trial rather than newly published articles because there is less variation in pageviews, and Mendeley saves for older articles, which made it more efficient to detect possible effects of the intervention. Articles were randomized to either the TrendMD arm (*n* = 200) or the control arm (*n* = 200) of the study and outcomes were measured at 4-week. The overall study design is presented in Fig. 1.

### Intervention

**Fig. 2:**
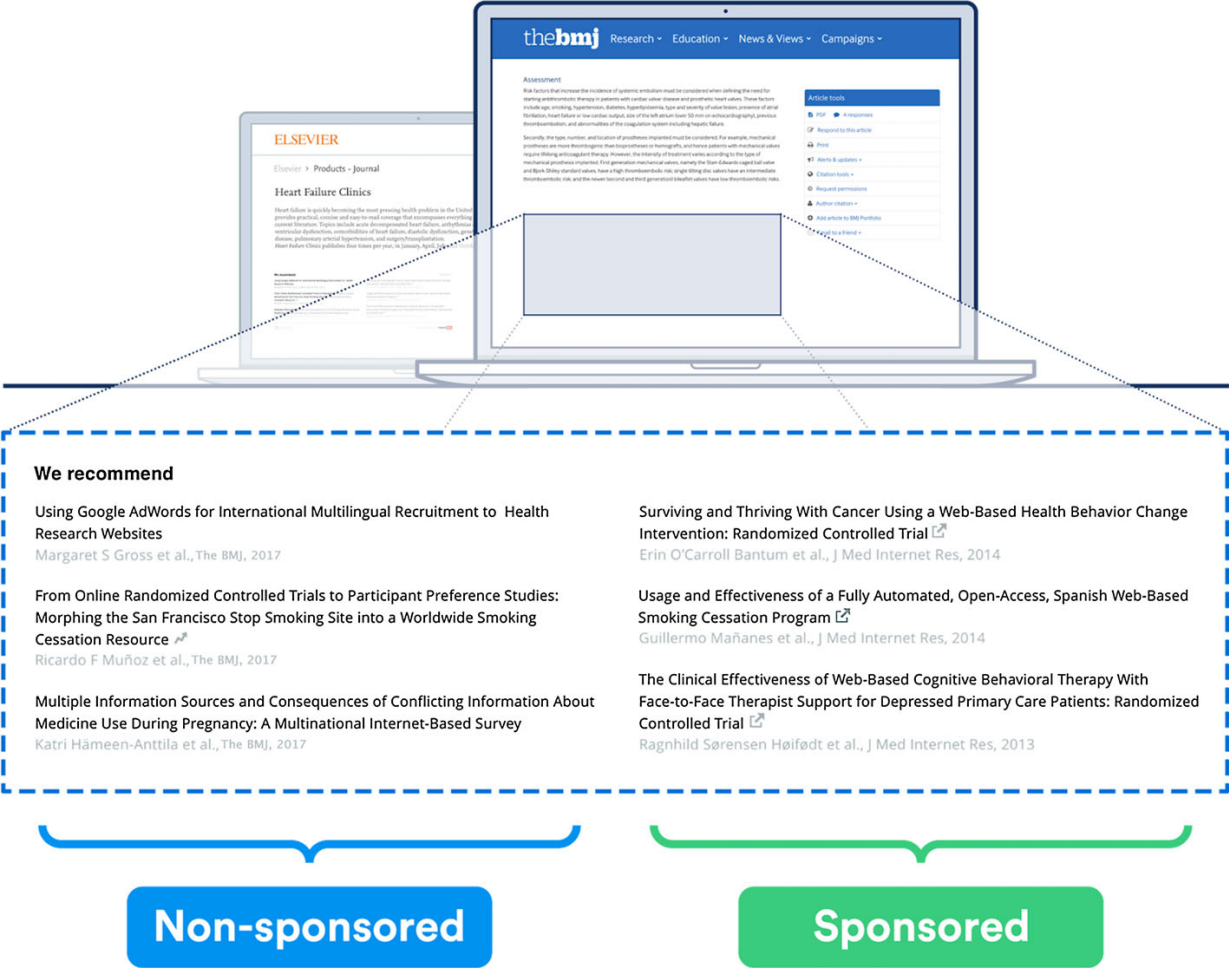
How TrendMD is displayed on a journal in the TrendMD Network

For background, TrendMD (www.trendmd.com) is a cross-publisher article recommendations and distribution platform that is embedded on over 3300 journals and websites from 300 publishers and seen by approximately 80 million readers per month (TrendMD Inc. 2017). Participating publishers use TrendMD to distribute their published article links within the article recommendations displayed on articles within their journals (non-sponsored recommendations) or on third-party journals within the TrendMD Network (sponsored recommendations) (Fig. 2). TrendMD’s content distribution model is benchmarked to similar services in the consumer web, where the leading networks Outbrain (www.outbrain.com) and Taboola (www.taboola.com) generate the “From the web” and “You may like” recommendations seen alongside the content on many popular websites like CNN or New York Times (Kudlow et al. 2016). Among the chief possible reasons why TrendMD may be an effective distribution channel, is that TrendMD is recommending articles to readers directly in context, when they are reading other scholarly material (Fig. 2).

**Fig. 3:**
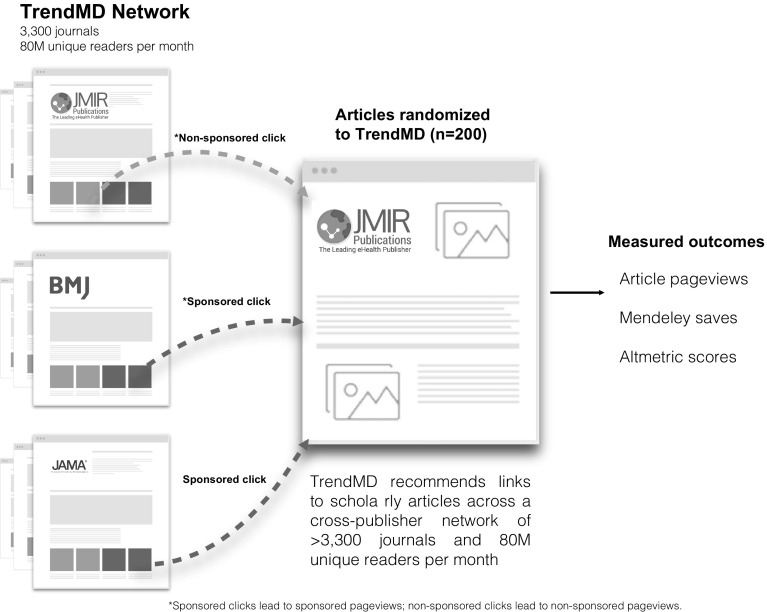
How TrendMD works

The intervention consisted of exposure of original articles published in JMIR in the TrendMD Network between November 14 and December 14 2016. Articles included in the TrendMD Network are displayed as recommended article links (Kudlow et al. 2016). Links to articles randomized to TrendMD were displayed as both non-sponsored recommended links on online journals published by JMIR Publications Inc. (*n* = 14) and sponsored recommended links on third-party publications participating in the TrendMD Network (*n* = 3300 journals, 80 million readers per month as of November 14, 2016). The frequency of both non-sponsored and sponsored article link placements are determined by a relevancy score based on the following: relatedness (i.e. keyword overlap), collaborative filtering (similar to Amazon’s “people who bought this item also bought that item”), and user clickstream analysis (the Netflix approach, basing recommendations on the users’ interests expressed through their online history) (Kudlow et al. 2014, 2016). As a result of the relevancy scoring system, some articles randomized to TrendMD were seen more often (i.e. accrued more link impressions) than others in the TrendMD Network. The publisher was charged a cost-per-click fee when their sponsored article links were clicked. The publishers sponsored links are displayed in the TrendMD Network so long as they are relevant and the publisher’s account balance is greater than $0. The 200 articles randomized to TrendMD received a maximum total budget of $500 at a cost-per-click of $0.4 USD for 1250 sponsored TrendMD clicks. The actual amount spent by the publisher was $421.60 (of the total allocated budget of $500) over the 4-week trial (1054 sponsored clicks received by the 200 article randomized to TrendMD at $0.4 cost per click). There is no fee for clicks on the publisher’s non-sponsored links displayed in the JMIR journals. A summary of how TrendMD works is presented in Fig. 3.

### Control

Articles randomized to control (*n* = 200) received no promotion in the TrendMD Network or any other social media networks. Traffic received by articles randomized to control was by organic means only (e.g. Google, Google Scholar, PubMed, etc.).

### Primary outcome (Table 1)

The primary outcome compares the mean Mendeley reader counts (i.e. saves) over the 4-week trial for articles randomized to TrendMD versus control. A Mendeley reader is counted when an article has been saved to a Mendeley user library account (Mendeley). Mendeley saves were selected because this metric has been shown to correlate to future article citations (Ebrahimy et al. 2016; Thelwall and Wilson 2016). Mendeley reader counts were abstracted through the Altmetric Explorer and cross-referenced with the Mendeley API (Mendeley).

**Table 1:** Definitions of primary and secondary outcomes

Metric	Definition	Collection method
Mendeley reader count (i.e. saves)	A Mendeley reader is counted when an article has been saved to a Mendeley user library account	Altmetric Explorer, cross-referenced with Mendeley API
Total pageviews	An instance of a page being loaded in a browser. Pageviews is a metric defined as the total number of pages viewed. For articles randomized to TrendMD, total pageviews is equal to the sum of organic pageviews and TrendMD total pageviews	JMIR Google Analytics account
Organic pageviews	Organic pageviews are equal to total pageviews for articles randomized to control. For articles randomized to TrendMD, organic pageviews is equal to the difference between total pageviews and TrendMD total pageviews (i.e. a measure of total pageviews after subtracting TrendMD total pageviews)	JMIR Google Analytics account
TrendMD total pageviews	The total number of pageviews from TrendMD. It is equal to the sum of TrendMD non-sponsored and sponsored pageviews	JMIR Google Analytics account, cross-referenced with JMIR TrendMD Analytics Dashboard
TrendMD non-sponsored pageviews	TrendMD pageviews from clicks on JMIR non-sponsored article links displayed in TrendMD recommendations on JMIR Inc. journals. For the purpose of this investigation, we assume that 1 TrendMD non-sponsored click leads to 1 TrendMD non-sponsored pageview	JMIR Google Analytics account, cross-referenced with JMIR TrendMD Analytics Dashboard
TrendMD sponsored pageviews	TrendMD pageviews from clicks on JMIR sponsored article links displayed in TrendMD recommendations on participating publisher sites (3300 as of November 14, 2016). For the purpose of this investigation, we assume that 1 TrendMD sponsored click leads to 1 TrendMD sponsored pageview	JMIR Google Analytics account, cross-referenced with JMIR TrendMD Analytics Dashboard
Altmetric score	Altmetric.com collects, scores, and weights mentions of academic articles on social media platforms (Twitter, Facebook, etc.), news outlets, and, blog posts. The Altmetric score is mutually exclusive to Mendeley saves and pageviews. The Altmetric score is a proprietary metric from Altmetric.com	Altmetric Explorer
Bounce rate	The percentage of single-page visits (i.e. visits in which the person left a website from the entrance page without interacting with the page)	JMIR Google Analytics account
Session	The period time a user is actively engaged with website. All usage data (screen views, events, ecommerce, etc.) is associated with a session	JMIR Google Analytics account
Pages per session	The number of pages viewed during a session	JMIR Google Analytics account
Session duration	The length of a session	JMIR Google Analytics account

### Secondary outcomes (Table 1)

Mean differences in total pageviews, organic pageviews, and Altmetric scores for articles randomized to TrendMD versus control were selected as secondary outcomes. Altmetric scores were included as a secondary outcome because they do not include Mendeley saves or article pageviews (Altmetric 2015, 2016). In addition we collected engagement metrics, which include bounce rates, mean time spent on article pages, and pages per session for readers who clicked on TrendMD sponsored links versus organic pageviews of control articles. See Table 1 for definitions of outcomes collected. Article pageview and engagement data were abstracted through JMIR Google Analytics account, including HTML and PDF pageviews. Altmetric scores data were abstracted through Altmetric Explorer.

### Statistical methods

We performed an a priori power calculation to determine necessary sample size. Based on our groups’ prior research (Kudlow et al. 2014, 2016), we assumed that both primary and secondary outcomes had a log-normal distribution. We assumed that the 4-week difference in mean Mendeley article saves between the control group and TrendMD would be 5, with a standard deviation of 20. Therefore, assuming a log-normal distribution for 4-week mean Mendeley saves, an effect of our intervention could be detected at 80% power using a 2-sided (alpha = 0.05) by a sample size of 195 papers in each group (Kadam and Bhalerao 2010).

Baseline characteristics of articles were tabulated and compared on log-transformed data across randomized study arms using the 2-sample *t* test for independence. We categorized articles by publication date and used the Chi-square to test for independence. The primary analysis compares 4-week mean Mendeley saves by 2-sample *t* test on the log-transformed data. Our secondary analysis also uses the 2-sample *t* test on the log-transformed data to compare means in pageviews, engagement metrics, and Altmetric scores between TrendMD and control. We calculated the effect size using Cohen’s *d* (Cohen 1977). Lastly we performed a multivariate regression analysis using TrendMD sponsored and non-sponsored pageviews, as well as organic pageviews to predict a change in Mendeley saves over the 4-week trial. R version 3.3.2 was used to complete the statistical analysis.

## Results

### Baseline characteristics (Table 2)

Overall, 400 articles were randomized: 200 to the TrendMD arm and 200 to the control arm. A Kolmogorov–Smirnov test of the pageview (*p* = 0.26), Mendeley saves (*p* = 0.15), and Altmetric score (*p* = 0.57) data confirmed that the distributions were log-normal within the control and TrendMD arms. As shown in Table 2, there were no differences in article total pageviews (*p* = 0.40), Mendeley saves (*p* = 0.35), Altmetric scores (*p* = 0.46), or publication date (*p* = 0.92) at the study onset for articles randomized to TrendMD versus control.

**Table 2:** Study sample characteristics by trial arm

Metrics	TrendMD (*n* = 197)		Control (*n* = 198)		*p* value (2-sample *t* test)
	Mean (SD)	Median	Mean (SD)	Median	
Mendeley saves	20.7 (14.4)	19	21.3 (17.7)	17	0.35
Altmetric score	26.8 (42.6)	14	27.5 (57.3)	14	0.46
Total pageviews	884.0 (862.5)	678.3	979.4 (1319.1)	627.1	0.40

Publication date			*p* value (Chi square test)
October 2014–April 2015	63	60	0.92
May 2015–November 2015	74	78	
December 2015–May 2016	60	60	

### Primary outcome (Table 3)

**Fig. 4:**
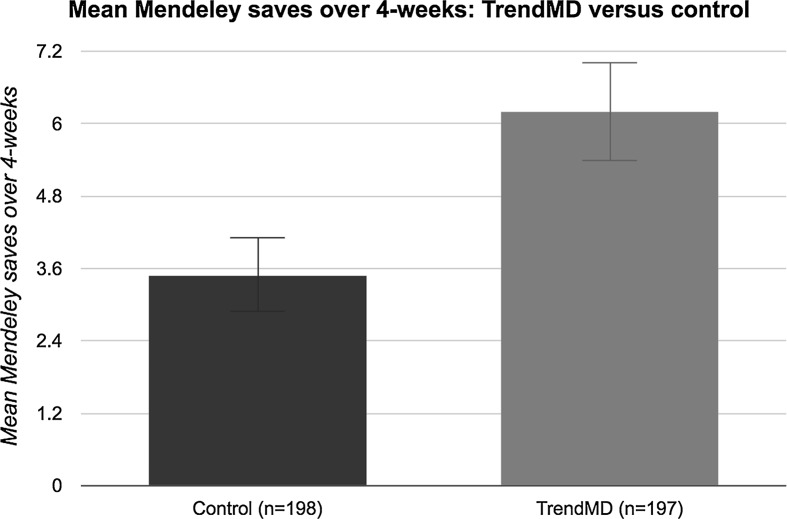
Mean Mendeley saves of TrendMD versus control over 4-week

**Fig. 5:**
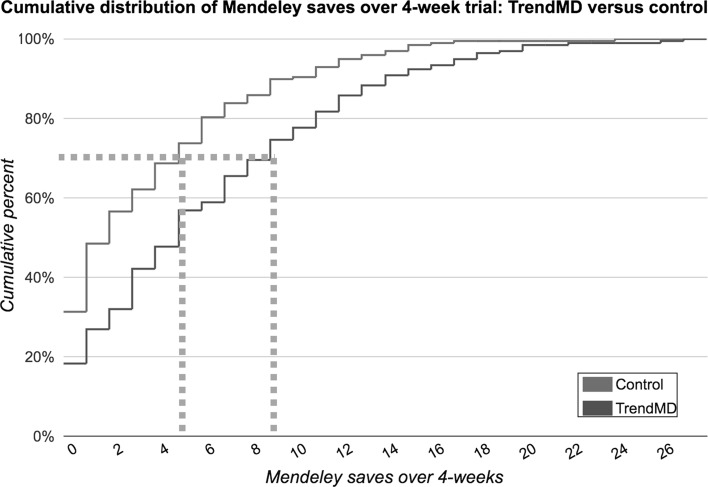
Cumulative distribution of Mendeley saves over 4-week trial: TrendMD versus control. Right shift of TrendMD line (*red*) indicates that a higher percentage of articles in the TrendMD arm had an increased number of Mendeley saves versus control (*blue line*). For example, as indicated by the *grey dotted line*, 70% of articles randomized to TrendMD had 9 Mendeley saves or less, whereas control articles had 5 Mendeley saves or less, over the 4-week trial. (Color figure online)

Articles randomized to the TrendMD arm received a 77% increase in mean saves on Mendeley relative to control over the 4-week trial. The mean Mendeley saves for articles randomized to TrendMD was 6.2 (median = 5; SD = 5.7), compared to 3.5 (median = 2; SD = 4.3) for articles randomized to control (Fig. 4). The difference in mean Mendeley saves for articles randomized to TrendMD versus control was 2.7 saves, 95% CI (2.63–2.77). The effect size of TrendMD on article Mendeley saves was moderate (Cohen’s *d* = 0.53) and statistically significant (*p* < 0.01). The cumulative distribution of article Mendeley saves over the 4-week trial is shown in Fig. 5.

**Table 3:** Primary and secondary outcomes

	TrendMD (*n* = 197)		Control (*n* = 198)		Mean difference (95% confidence interval)	*p* value (2-sample *t* test)	Cohen’s *d*
	Mean (SD)	Median	Mean (SD)	Median			
Mendeley saves	6.2 (5.8)	5	3.5 (4.3)	2	2.7 (2.63–2.77)	<0.01	0.53
Altmetric score	0.44 (2.0)	0	0.16 (0.54)	0	0.28 (0.26–0.30)	0.031	0.19
Total pageviews	35.9 (27.1)	30	18.4 (28.1)	13	17.5 (17.11–17.89)	<0.01	0.64
Organic pageviews	25.2 (24.4)	17	18.4 (28.1)	13	6.8 (6.43–7.17)	<0.01	0.26
TrendMD total pageviews	10.8 (8.5)	9	N/A				
TrendMD non-sponsored pageviews	5.5 (5.4)	4	N/A				
TrendMD sponsored pageviews	5.4 (5.9)	4	N/A

### Secondary outcomes (Table 3)

#### Pageviews

**Fig. 6:**
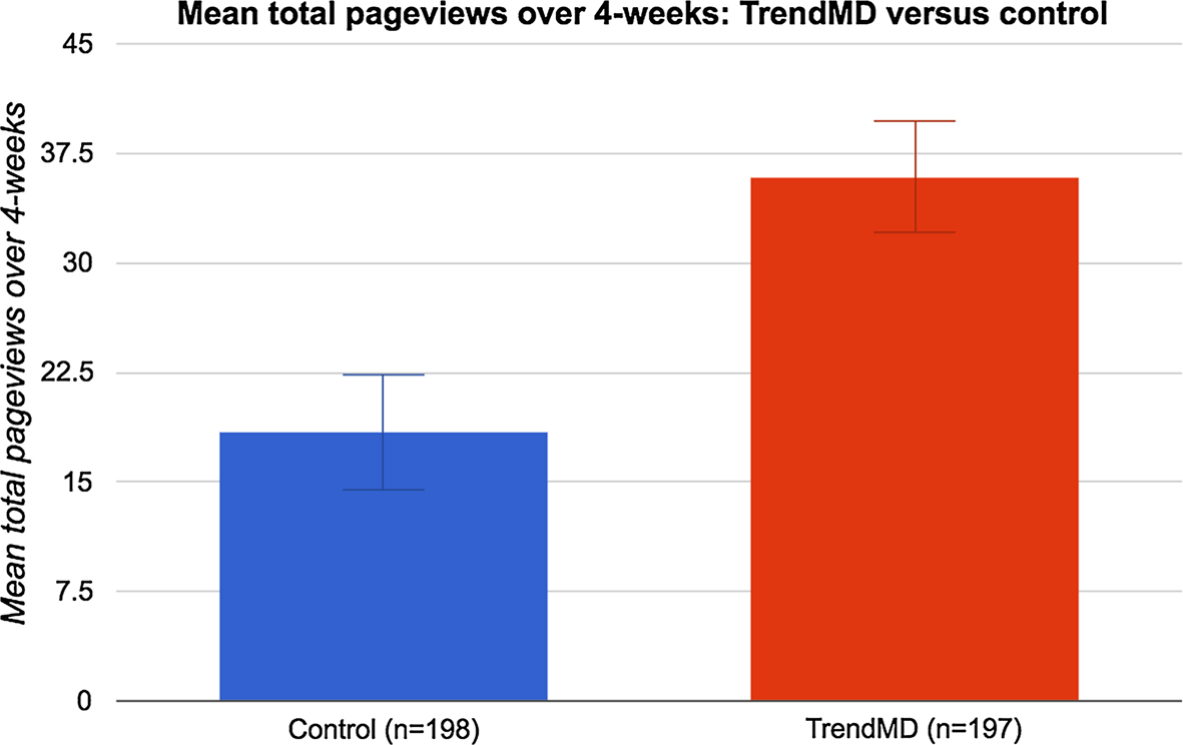
Mean total pageviews: TrendMD versus control

Articles randomized to the TrendMD arm received a 95% increase in mean total pageviews relative to control over the 4-week trial. The mean total pageviews for articles randomized to TrendMD was 35.9 (median = 30; SD = 27.1), whereas control articles had a mean of 18.4 total pageviews (median = 13; SD = 28.1). The difference in mean total pageviews for articles randomized to TrendMD versus control was 17.5 pageviews, 95% CI (17.11–17.89) (Fig. 6). The effect size of TrendMD on total pageviews was moderate-to-large (Cohen’s *d* = 0.64) and statistically significant (*p* < 0.01).

**Fig. 7:**
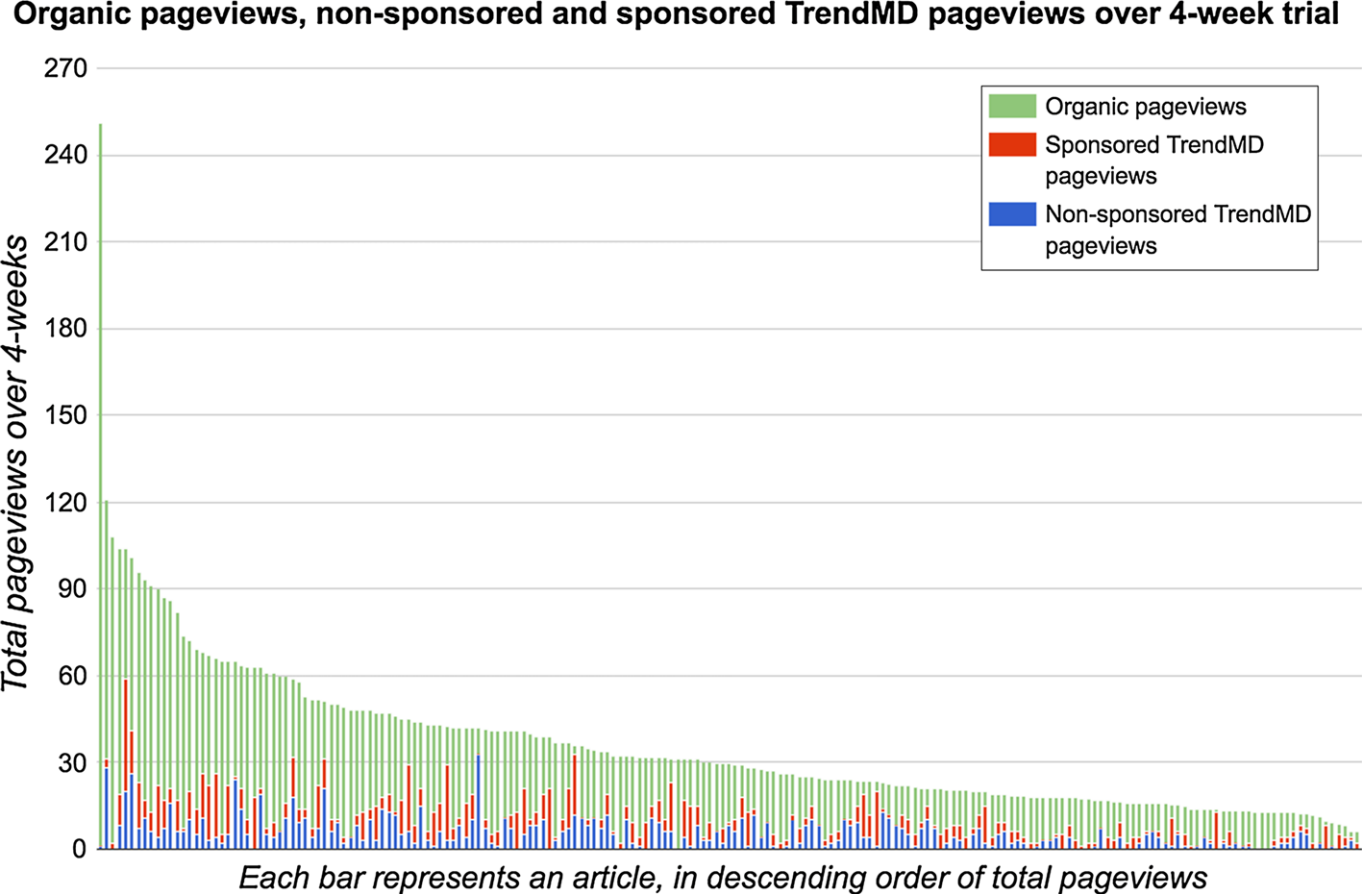
Organic pageviews, non-sponsored and sponsored TrendMD pageviews over 4-week trial. Total pageviews = organic pageviews + TrendMD non-sponsored pageviews + TrendMD sponsored pageviews

Thirty-percent of the mean total pageviews (mean = 10.8; median = 9; SD = 8.5) of articles randomized to the intervention were due to clicks on either TrendMD non-sponsored or sponsored article links. TrendMD non-sponsored clicks lead to a mean of 5.5 pageviews (median = 4; SD = 5.4) and TrendMD sponsored clicks lead to a mean of 5.4 pageviews (median = 4; SD = 5.9). Figure 7 is a histogram of the traffic received by articles randomized to TrendMD. In Table 4, we examined the top 20 publishers and journals in the TrendMD Network who sent traffic to articles randomized to TrendMD through sponsored links.

**Table 4:** Top referring publishers and journals

Publisher name	Journal name	TrendMD non-sponsored pageviews	TrendMD sponsored pageviews	CTR (%)
BMJ Group	The BMJ	113	25,111	0.45
American Diabetes Association	Diabetes Care	93	28,182	0.33
BMJ Group	Br J Sports Med	83	18,864	0.44
American Academy of Pediatrics	Pediatrics	71	16,905	0.42
American Society of Clinical Oncology	J Clin Oncol	68	14,167	0.48
Proceedings of the National Academy of Sciences	Proc Natl Acad Sci U S A	62	22,963	0.27
American Society for Nutrition	J Nutr	51	15,000	0.34
American Society for Nutrition	Am J Clin Nutr	33	7174	0.46
BMJ Group	BMJ Open	30	7143	0.42
American Psychiatric Association	American Journal of Psychiatry	22	3929	0.56
Co-Action Publishing	Research in Learning Technology	18	10,000	0.18
Royal Society	Philosophical Transactions B	12	5455	0.22
Boston Globe	STAT News	11	2037	0.54
BMJ Group	Tob Control	10	2703	0.37
Medgadget	Medgadget	10	1695	0.59
Elsevier	Clinics in Sports Medicine	6	2500	0.24
European Respiratory Society	European Respiratory Journal	5	2174	0.23
The Journal of Bone and Joint Surgery	J Bone Joint Surg Am	5	1042	0.48
Other	Other	351	90,000	0.39
Total		1054	277,041	0.38

**Fig. 8:**
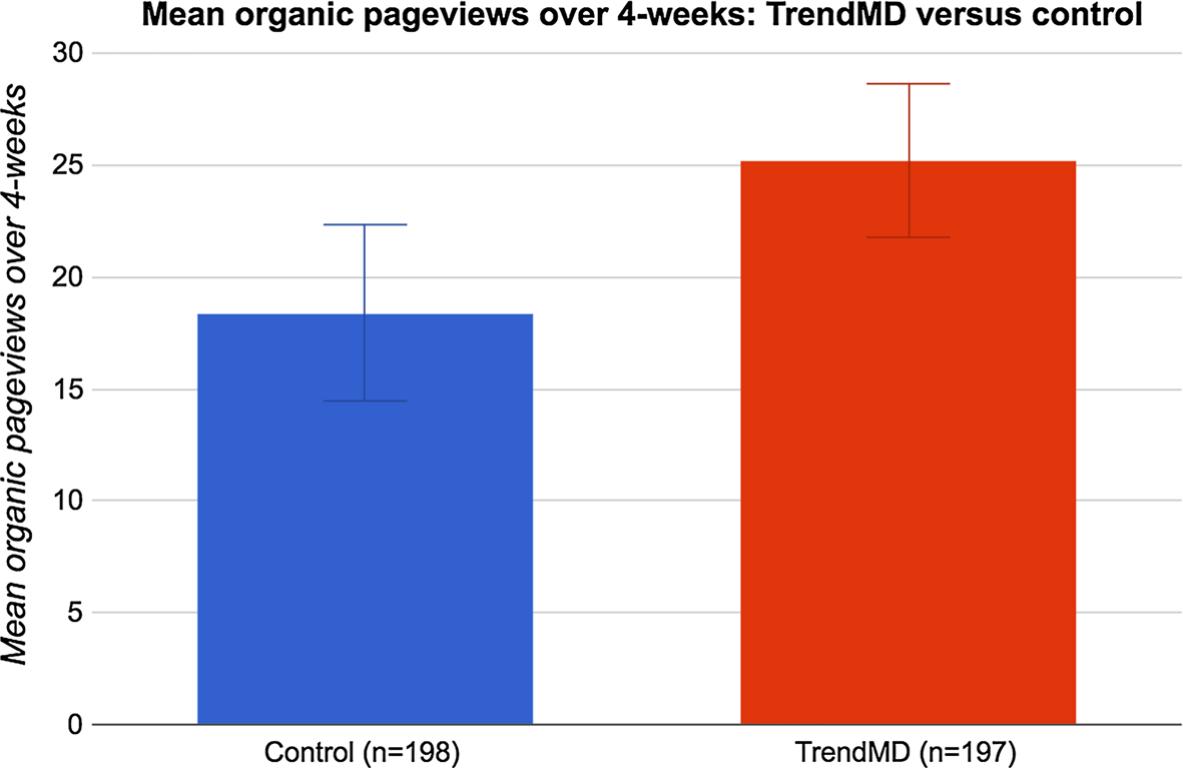
Mean organic pageviews: TrendMD versus control

Lastly, articles randomized to TrendMD received a 37% increase in mean organic pageviews relative to control over the 4-week trial (Fig. 8). The mean organic pageviews for articles randomized to TrendMD was 25.2 (median = 17; SD = 24.4), whereas control articles had a mean of 18.4 organic pageviews (median = 13; SD = 28.1). The difference in mean organic pageviews for articles randomized to TrendMD versus control was 6.8 pageviews, 95% CI (6.43–7.17). The effect size of TrendMD on organic pageviews was small (Cohen’s *d* = 0.26) and statistically significant (*p* < 0.01).

#### Altmetric scores

TrendMD had a small and statistically significant effect on Altmetric scores. The mean Altmetric score for articles randomized to TrendMD was 0.44 (median = 0; SD = 2.0), whereas articles randomized to control had a mean of 0.16 (median = 0; SD = 0.54). The difference in mean Altmetric scores for articles randomized to TrendMD versus control was 0.28, 95% CI (0.26–0.30). The effect size of TrendMD on Altmetric scores was small (Cohen’s *d* = 0.19) and was a statistically significant (*p* = 0.031).

#### Engagement metrics (Table 5)

People who visited JMIR by TrendMD sponsored links to articles in the intervention group were more engaged when compared to those who accessed control articles via organic means (i.e. PubMed, Google Scholar, Google, etc.). This is evidenced by the fact that in comparison to who viewed control articles, those who accessed the articles randomized to TrendMD had lower bounce rates, and visited a greater number of pages per session (Table 5). There however was no statistical difference in mean session duration between the two groups.

**Table 5:** Engagement metrics for TrendMD sponsored pageviews versus control organic pageviews

	TrendMD: sponsored visitors (*n* = 1054)	Control: organic visitors (*n* = 3642)	*p* value (2-sample *t* test)
	Mean (SD)	Mean (SD)	
Bounce rate	9.79% (28.7%)	41.4% (31.2%)	<0.01
Pages per session	4.82 (2.17)	2.35 (1.93)	<0.01
Session duration	0:01:12 (0:00:58)	0:01:04 (0:01:09)	0.237

### Multivariate regression

We completed a multivariate regression model for the effects of TrendMD sponsored and non-sponsored pageviews, as well as organic pageviews on Mendeley article saves over the 4-week trial. The parameters of the model include:

- MS: Mendeley article saves.
- TS: TrendMD sponsored pageviews.
- TN: TrendMD non-sponsored pageviews.
- O: Organic pageviews.

The linear regression model can be expressed as:

$$MS = 0.42*TS + 0.381*TN + 0.057*O + \varepsilon \;({\text{Alexopoulos}}\;2010)$$

**Fig. 9:**
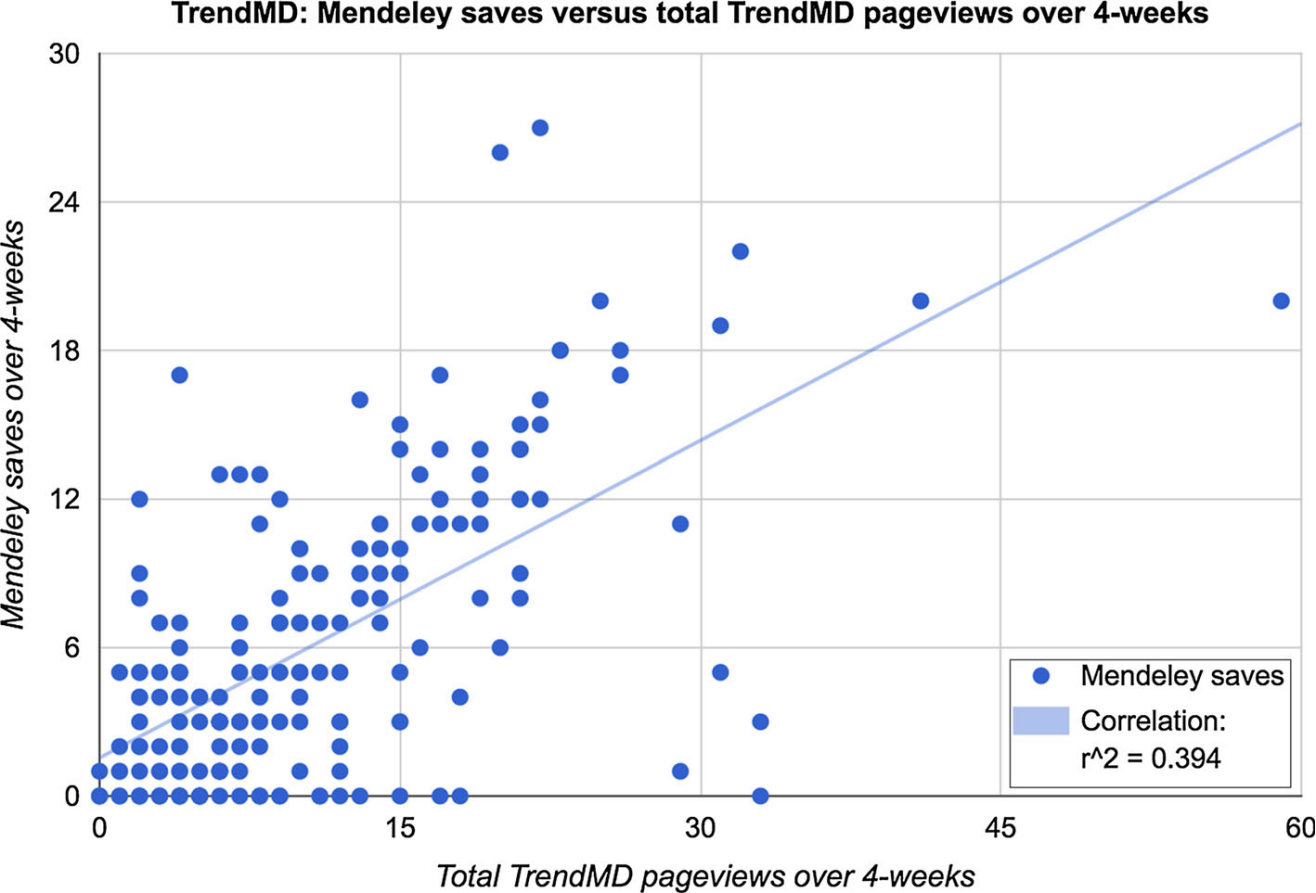
Mendeley saves versus total pageviews driven by TrendMD over 4-week. *R* squared of 0.394 indicates that 39.4% of the variation of Mendeley saves can be attributed to TrendMD. Spearman’s rho if 0.60 indicates a strong relationship between TrendMD pageviews and Mendeley saves, over the 4-week trial (Cohen’s *d* = 0.53)

Our model was a good fit; both TrendMD driven pageviews and organic pageviews predicted 46% of the variation in Mendeley saves. All predictor variables in the model were statistically significant (*p* < 0.0001). Shown in Fig. 9 is a correlation graph between TrendMD article pageviews and article saves on Mendeley (Spearman’s rho *r* = 0.60; *r* squared = 0.394).

**Fig. 10:**
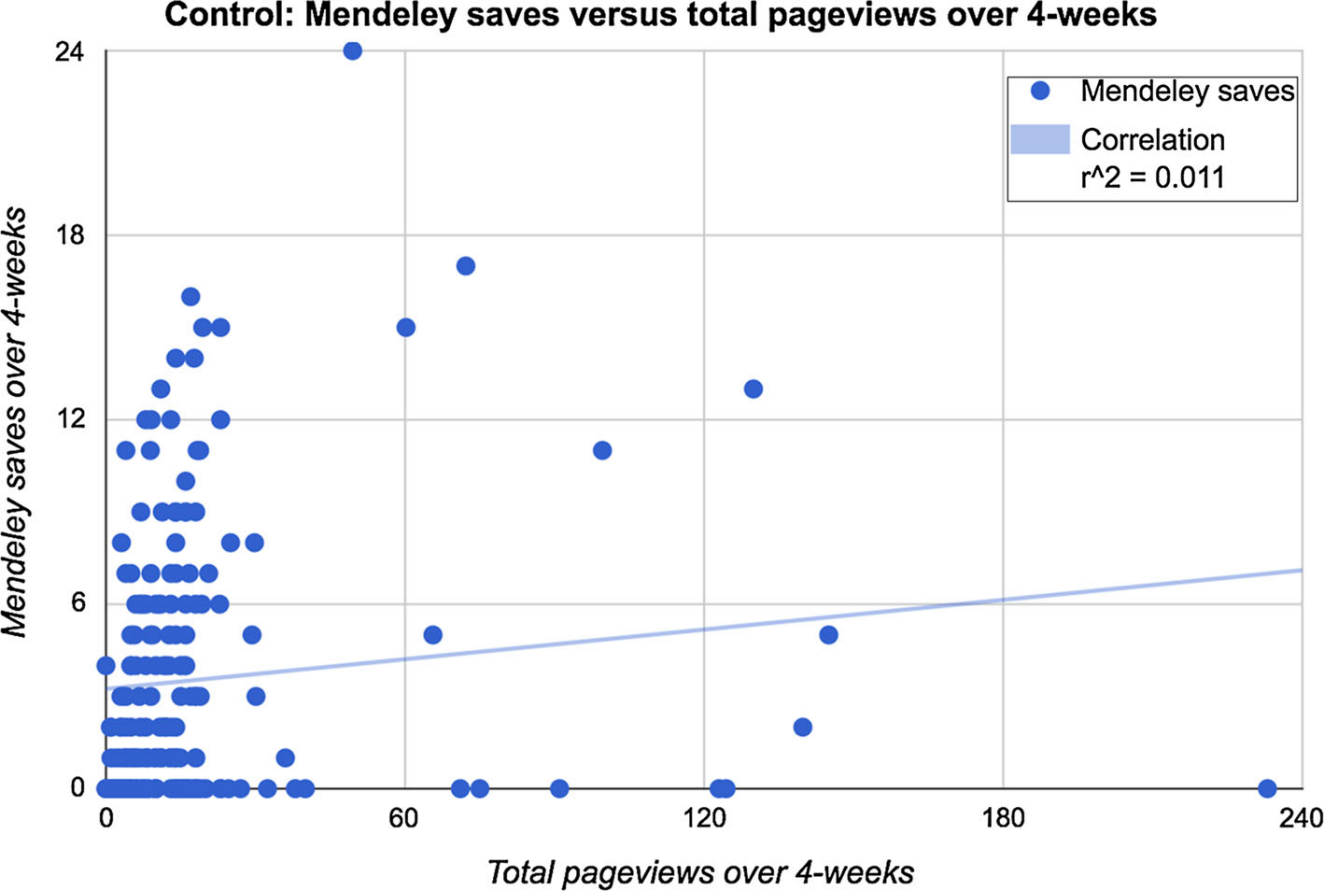
Articles randomized to control: Mendeley saves versus total pageviews over 4-week. *R* squared of 0.01 indicates that total pageviews predicted 1% of the variation in Mendeley saves of articles randomized to control group

Since we found a statistically significant difference between mean organic pageviews of articles randomized to TrendMD versus control, we completed a secondary regression model to examine if pageviews through TrendMD predicted organic pageviews. Though there was a relationship between TrendMD pageviews and organic pageviews (Beta = 0.503; *p* < 0.0001), TrendMD pageviews only predicted 3.0% (*r* squared = 0.03) of the variation in organic pageviews. However, when we removed 2 outlying articles which received greater than three-times the standard deviation from the mean (10.2196/jmir.3652, 10.2196/jmir.4052), TrendMD pageviews predicted 12.2% of the variation in organic pageviews (Beta = 0.722; *p* < 0.0001). Lastly, we found no correlation between total article pageviews and Mendeley saves for articles randomized to control over the 4-week trial (Fig. 10; *R* squared control 0.011).

## Discussion

This is the first rigorous investigation to show how an online cross-publisher distribution channel (TrendMD) can be used to increase scholarly article Mendeley saves, pageviews, and, Altmetric scores. TrendMD had statistically significant effects on all outcomes measured, with the strongest effect size on pageviews, followed by Mendeley saves, organic pageviews, and a very small effect size on Altmetric scores. Our study significantly adds to the relatively scant corpus of literature that examines the efficacy of online strategies to distribute peer-reviewed content. Prior research has yielded inconclusive results as to whether online distribution strategies, such as social media can enhance pageviews and/or impact of scholarly literature (Fox et al. 2014, 2016b; Dixon et al. 2015; Thoma et al. 2015; Hand et al. 2016). These data address an important unmet need of scholarly content providers for evidenced-based methods to effectively distribute individual peer-reviewed articles.

These findings are consistent with our prior findings, in which we showed how cross-publisher distribution via TrendMD lead to a 49% increase in weekly article pageviews relative to baseline traffic over a 3-week period (Kudlow et al. 2016). Our prior findings were limited however by the crossover design (i.e. we had no control group), relatively small TrendMD Network size (1100 and 12 million readers per month), and outcome measure of pageviews. The current study addresses these limitations through the randomized controlled trial study design, larger TrendMD cross-publisher network (>3300 journals and websites 80 million readers per month), and more robust impact measure of differences in mean Mendeley saves.

Several findings were interesting to note from the data presented herein. One key finding was the statistically significant difference in mean organic pageviews between TrendMD and control. This suggests that individuals arrived at articles randomized to TrendMD more frequently via the Internet compared to control articles. One possible explanation is that discovery of articles via TrendMD lead to secondary effects, which lead to individuals visiting articles randomized to TrendMD more frequently. Some of these secondary effects could be readers coming back to articles independently (e.g. saving them as bookmarks on an Internet browser and visiting it later), sharing articles with their colleagues over email, or spreading via word of mouth. Though we have no methods of directly measuring attribution of the additional organic pageviews, our data indicates that TrendMD visitors were more engaged when compared to control. This could indicate that more secondary effects, such as more sharing of articles took place. Increased independent return of visitors and sharing is also supported by the fact that TrendMD pageviews were correlated to organic pageviews. Another secondary effect could have been that TrendMD lead to enhancements to Search Engine Optimization (SEO) on Google (i.e. TrendMD articles ranked higher in Google Search results due to more backlinks from TrendMD recommended links) (Killoran 2013). Therefore, enhancements to SEO could have lead to more organic pageviews in the TrendMD group versus control.

Another interesting finding was that the standard deviation in organic pageviews was lower, and the median organic pageviews was higher in articles randomized to TrendMD versus control. This indicates that TrendMD lead to more evenly distributed visibility of articles. These data could indicate that TrendMD may encourage discoverability of articles that aren’t normally seen and/or not normally searched for. The general rule is scholars tend to read current articles more often than older articles, as part of their strategy for keeping up to date (Tenopir et al. 2012). However, electronic availability of articles and better search technology has prompted more reading of older articles by U.S. science faculty (Tenopir et al. 2009; Acharya et al. 2014). Based on the data collected herein, TrendMD more evenly distributed visibility of articles, even beyond the effects of electronic publishing and enhancements in search technology.

Strengths of this study include the rigorous trial design that was adequately powered for our primary outcome (mean Mendeley saves). The primary outcome was objective and unbiased between the control and intervention arms. However, some limitations warrant mention. Firstly, authors PK, MC, DT, DBD, AR, and, GE all have conflicts of interest with the results presented herein. Risk of bias, however, was mitigated by our single-blinded, randomized-controlled trial design as well as inclusion of authors, RM, AS, and, AR, who do not have a conflict of interest. Furthermore, this study was completed with articles published in an Open Access journal with a potentially technology savvy audience. Replication is needed in different academic disciplines and including closed-access content to determine if these data are generalizable. Notwithstanding, the referring publisher data indicate that visitors came from journals that publish content across academic disciplines, which suggests that these findings may be generalizable to other disciplines. Another limitation is that we did not capture any citation data. While prior research suggests that Mendeley saves are a robust predictor of future citations, we currently do not have any data to support that our increase in Mendeley usage will lead to future citations. Another possible limitation of this current investigation is length of the study; it’s possible that TrendMD’s effect size on Mendeley saves may saturate and diminish over a longer period of time. Future studies with a longer duration are planned to test the possibility of saturation effects of cross-publisher distribution via TrendMD on Mendeley saves. Lastly, our results were limited by the fact that no other online interventions, including social media, as well as single-publisher article recommendations were tested. Future studies are planned to test the effects of distribution of articles via both paid and unpaid social media channels, as well as single-publisher recommendations, in parallel with cross-publisher recommendations via TrendMD.

Notwithstanding, herein we show how a cross-publisher online distribution channel (TrendMD) can be used to increase scholarly article saves on Mendeley, pageviews, and, Altmetric scores. Replicated data has shown that Mendeley article saves correlate to future citations (Priem et al. 2012; Lin and Fenner 2013; Zahedi et al. 2014; Ebrahimy et al. 2016; Maflahi and Thelwall 2016; Thelwall and Wilson 2016; Li and Thelwall 2012). Therefore, while replication and further study are needed, TrendMD may be an online distribution channel that can be used to increase citations of scholarly articles.
